# Endothelial Differentiation G Protein‐Coupled Receptor 5 Plays an Important Role in Induction and Maintenance of Pluripotency

**DOI:** 10.1002/stem.2954

**Published:** 2019-02-07

**Authors:** Irina Neganova, Lewis Cotts, Peter Banks, Katja Gassner, Anvar Shukurov, Lyle Armstrong, Graham Ladds, Majlinda Lako

**Affiliations:** ^1^ International Centre for Life, Institute of Genetic Medicine Newcastle University Newcastle United Kingdom; ^2^ High Throughput Screening Facility Medical School Newcastle United Kingdom; ^3^ School of Mathematics and Statistics Newcastle University, Newcastle upon Tyne United Kingdom; ^4^ Department of Pharmacology University of Cambridge Cambridge United Kingdom

**Keywords:** hESCs/hiPSCs, Human somatic cell reprogramming, Genome‐wide RNAi screen, GPCR, EDG5, Cytoskeleton

## Abstract

Direct reprogramming of human somatic cells toward induced pluripotent stem cells holds great promise for regenerative medicine and basic biology. We used a high‐throughput small interfering RNA screening assay in the initiation phase of reprogramming for 784 genes belonging to kinase and phosphatase families and identified 68 repressors and 22 effectors. Six new candidates belonging to the family of the G protein‐coupled receptors (GPCRs) were identified, suggesting an important role for this key signaling pathway during somatic cell‐induced reprogramming. Downregulation of one of the key GPCR effectors, endothelial differentiation GPCR5 (*EDG5*), impacted the maintenance of pluripotency, actin cytoskeleton organization, colony integrity, and focal adhesions in human embryonic stem cells, which were associated with the alteration in the RhoA‐ROCK‐Cofilin‐PAXILLIN‐actin signaling pathway. Similarly, downregulation of *EDG5* during the initiation stage of somatic cell‐induced reprogramming resulted in alteration of cytoskeleton, loss of human‐induced pluripotent stem cell colony integrity, and a significant reduction in partially and fully reprogrammed cells as well as the number of alkaline phosphatase positive colonies at the end of the reprogramming process. Together, these data point to an important role of EDG5 in the maintenance and acquisition of pluripotency. Stem Cells *2019;37:318–331*

**Significance Statement**

Using a high‐throughput RNA interference screen, authors identified 22 effectors of somatic cell‐induced reprogramming, six of which belong to the G protein‐coupled receptor (GPCR) family. The present study describes a new role for the GPCR family member, endothelial differentiation GPCR5 (EDG5; sphingosine‐1‐phosphate receptor 2), whose downregulation in human embryonic stem cells (hESCs) or during the initiation period of reprogramming leads to abrogation of the pluripotent stem cell colony formation because of the defects in cytoskeleton organization and focal adhesions. Together, the data provide for the first time substantive evidence for EDG5 as a critical GPCR for the maintenance of pluripotency in hESC and successful reprogramming of human fibroblasts to human‐induced pluripotent stem cells.

## Introduction

Direct reprogramming of human somatic cells by ectopic expression of *OCT4, SOX2, KLF4*, and *c‐MYC* (OSKM) transcription factors results in generation of human‐induced pluripotent stem cells (hiPSCs), which are similar to human embryonic stem cells (hESCs) in many of their properties 1. Human iPSCs have been generated from various cell types 2, 3, 4 and have a great potential for regenerative medicine, because they enable the derivation of patient‐specific pluripotent cells and serve as a platform for stem‐based research, disease modeling, and drug discovery/repurposing 5, 6, 7, 8, 9. Despite extensive research toward understanding of the reprogramming process, the underlying mechanisms are not fully understood 10, 11, 12, hindering their effective application in clinical studies 13. A number of molecular and cellular barriers of reprogramming have been identified to date 14, 15, 16, resulting in an overall 2%–5% efficiency, thus indicating that the majority of cells are unable to complete reprogramming toward pluripotency 17, 18, 19.

Pluripotency induction during reprogramming occurs in discrete stages (initiation, maturation, and stabilization) and is characterized by specific alterations in the cellular transcriptome, epigenome 20, 21, 22, and stage‐specific modulation of various signaling pathways some of which have been recently elucidated in our recent publications 17, 18. Chemical inhibition of glycogen synthase kinase 3 23, transforming growth factor β (TGF‐β) signaling 23, 24, and inhibition of mitogen‐activated protein kinase (MAPK) signaling promote early stages of reprogramming, whereas the inactivation of Rb tumor suppressor promotes reprogramming and increases its efficiency 25. Activation of phosphoinositide3‐kinase (PI3K)‐AKT signaling, and focal adhesion (FA) as well as regulation of actin cytoskeleton, is required during the transition of fibroblasts to the pluripotent state 26.

To identify novel regulators of reprogramming, we developed a high‐throughput RNA interference (RNAi) screening assay. This strategy allowed us to perform knockdown of 784 members of the different kinases and phosphatases at the initiation stage of reprogramming. We identified 90 reprogramming candidates: 68 repressors and 22 activators, among which 76 were novel. Importantly, our list included previously recognized candidates in human (MPP3, TGFBR1, BUB1B, BMPR2, AKT1, NME5, ROCK2, RPS6KB2, TESK1, BMPR2, MELK, and SPHK2) and mouse cells (Act1, Acvr11, Tgfbr1, and Rps6kb2) 11, 15, 27, 28, 29. Among the top effectors, three members of the G protein‐coupled receptors (GPCRs) family, namely GPR42, GPR20, and endothelial differentiation GPCR5 (EDG5) were identified. In addition, three other GPCRs, GPR123, GPR116, and GPR37L1 were identified in our screen as potential reprogramming effectors.

There are more than 800 GPCRs in the human genome, making it the largest receptor superfamily of cell‐surface signaling proteins that bind extracellular ligands and transduce signals into cells via heterotrimeric GTP‐binding (G) proteins. The human GPCR superfamily is divided in five distinct families: rhodopsin, secretin, glutamate, adhesion, and frizzled receptors. G‐proteins, composed of α, β, and γ subunits, are central components of the primary mechanisms used by cells to respond to diverse extracellular stimuli. Most GPCRs activate one or multiple G‐alpha (Gα) subunits, which can be subdivided into four major families: Gi, G12/13, Gs, and Gq. Once activated, G protein subunits modulate secondary messenger release and activate various downstream intracellular signaling pathways (including adenylyl cyclase, phospholipases A2, C (PLC), and D, calcium mobilization, MAPK, extracellular signal‐regulated kinases‐1/2 ERK[1/2], PI3K, and the activation of small GTPases such as Rho and Rac), leading to regulation and adaptation of cellular functions to an external stimulus.

Knockdown of some GPCRs and/or components of their signaling pathways (Gs subunit, for example) are embryonic lethal or associated with significant developmental anomalies in mice and humans 30. However, despite the fundamental role of GPCRs and G proteins, their function during development and reprogramming process remains largely unexplored. A recently published report indicates that 116 GPCRs are expressed in hESCs, with 39 of these being upregulated and 20 downregulated during somatic reprogramming to hiPSC. Furthermore, 106 GPCRs are upregulated in hESC and hiPSC when compared to somatic cells, suggesting a putative role in maintenance of pluripotency or early differentiation 31, 32. This is further supported by the published reports, showing the Gs pathway and cAMP to be important contributors of mouse and human ESC self‐renewal and pluripotency 33, 34.

Two known positive regulators of pluripotency in hESCs, sphingosine‐1‐phosphate (S1P), and lysophosphatidic acid 35, 36, act via a single subfamily of GPCRs, designated as endothelial differentiation gene (EDG) family. The five EDG receptors specific for S1P are coupled to overlapping yet distinct sets of intracellular signaling pathways. Recently *EGD5* was identified as a novel gene required for stem cell pluripotency 37. However, until now, there are no detailed studies dedicated to the role of this gene during reprogramming of somatic cells toward hiPSCs. We investigated the function of EDG5 in maintenance of pluripotency in hESC and during somatic cell‐induced reprogramming of human neonatal fibroblasts (HNFs). Our data indicate that EDG5 is important for the maintenance of colony morphology, organization of actin cytoskeleton and FAs, and the suppression of mesoendermal gene expression in hESC. Similarly, downregulation of *EDG5* during the initiation stage of reprogramming process resulted in loss of colony integrity, dysregulation of actin cytoskeleton, and a significant reduction in the number of pluripotent stem cell colonies. Together, our data provide for the first time, substantive evidence for EDG5 as a critical GPCR for the maintenance of pluripotency in hESC and successful reprogramming of human fibroblasts to hiPSCs.

## Materials and Methods

### hiPSCs Generation and Cell Culture

CytoTune‐iPS 2.0 Sendai reprogramming kit (A16517, Invitrogen, Fisher Scientific UK Ltd; Loughborough, UK) was used for IPSC derivation as described recently on a feeder‐free culture on a plates covered with Matrigel (Corning Matrigel Matrix, Life Sciences, hESC‐qualified, High Wycombe, UK) 17. HNFs were purchased from Lonza (Slough, UK) and were cultured as described earlier 17.

### RNA Interference

SMARTpool: siGENOME small interfering RNA (siRNA) for *EDG5* was purchased from Dharmacon (M‐004253‐02‐0005; Supporting Information Table S1). The siRNA mixture at final concentration of 10 nM was used for transfection with DharmaFECT1 Transfection reagent (Dharmacon, Cambridge, UK, T‐2001‐01) according to manufacturer's instructions with OPTI‐MEM reduced serum Media (31985‐062; Gibco, Dublin, Ireland) for the first 45 minutes of transfection. Then an equal volume of the mTeSR1Medium (STEMCELL Technologies, Cambridge, UK) was added to cells. Media was changed for mTeSR1 every day. As a control ON‐TARGET*plus* nontargeting control pool from Dharmacon (D‐001810‐10) was used.

### Western Immunoblotting

Protein extraction, Western blotting, and antibody/antigen complex detection were performed as published previously 17. NE‐PER nuclear and cytoplasmic extraction reagent (78835) from ThermoScientific was used for nuclear and cytoplasmic extraction according to manufacturer protocol. Densitometry analysis was performed using ImageQuant TL software (GE Healthcare Life Sciences). Glyceraldehyde‐3‐phosphate dehydrogenase (GAPDH) was used to normalize band intensities of proteins of interest.

Primary antibodies were from Cell Signaling: for phospho‐cofilin (Ser3) (77G2, 3313), for total Cofilin (D3F9; 5175), for GAPDH (14C10; 2118), for Phospho‐FAK (Tyr397; D20B1; 8556), for total FAK (3285), for p‐MLC2 (Ser19; 3671), for TESK1 (D4904), for NANOG (D73G4; 4903), and for p‐ERK Thr202/Tyr204 (D13.14.4E). Primary antibodies from Abcam (Cambridge, UK) were: for PAXILLIN (phospho Y118; ab194738), for total PAXILLIN (ab2264), for ROCK1 (ab58305), and for GAPDH (ab9485). Antibody for p‐LIMK2 (Thr505) was from Invitrogen (PA537630). Primary antibodies from Santa Cruz (Heidelberg, Germany) were: for EDG5 (E‐12; sc365963), for vinculin (H‐10; sc‐253360), and for GAPDH (sc‐47724). Antibody for E‐cadherin was from ThermoFisher Scientific (5H6L18; 701134).

### Immunocytochemistry and Confocal Microscopy

Immunocytochemistry was performed as before 18. Primary antibodies used in this study were anti‐TRA‐1‐60 FITC conjugate (Merck Millipore, Watford, UK), anti‐EDG5 (E‐12) sc‐365963, anti‐NANOG cell signaling 4903, anti‐p‐Cofilin (Ser3; 77G2; 3313) cell signaling, anti‐p‐FAK (Tyr397; D20B1; 8556) cell signaling, anti‐p‐PAXILLIN (Tyr1218) ab194738, anti‐E‐cadherin (Cell Signaling Technology, New England BioLabs Ltd., Hitchin, UK), and 4′,6‐diamidino‐2‐phenylindole was from ThermoFisher. Rhodamine phalloidin from Life Technologies (R415) was used for visualization of filamentous actin The images were acquired with a Nikon A1R laser scanning confocal microscope (Nikon; http://nikon.com) using a CFl Plan Apochromat VC ×20/0.75 objective as described 18.

### Quantitative Reverse Transcription Polymerase Chain Reaction

Cells were harvested, and the total RNA was extracted using TRIzol (Invitrogen, 15596–026), according to manufacturer's instructions. All steps were performed as described before 18. Samples were normalized using GAPDH. All DNA oligonucleotide sequences are listed in Supporting Information Table S1.

### Flow Cytometric Analysis

Cells were disassociated using Versene (EDTA; Lonza), washed with phosphate‐buffered saline (PBS), and fixed in paraformaldehyde (2% final concentration in PBS) at 37°C for 10 minutes. After washing with PBS, the cells were permeabilized with prechilled methanol (−20°C) and incubated at 4°C for 30 minutes, followed by a washing step. Cells (0.2–0.5 × 10^6^) were resuspended in a total volume of 200 μl PBS containing 1% bovine serum albumin and incubated with appropriate amounts of anti‐CD44‐BV421 (Catalog number 562890; BD Biosciences, Oxford, UK; 1:300 dilution) and anti‐TRA‐1‐60‐FITC (Catalog number FCMAB115F; Merck Millipore; 1:100 dilution) monoclonal antibodies for 1 hour on a shaker, plate in the dark at room temperature. Finally, samples were washed using BD FACS Lyse Wash Assistant (BD Biosciences) and immediately analyzed on a flow cytometer. Fluorescence‐activated cell sorting (FACS) analysis was performed using BD FACS Canto II flow cytometer with FACSDiva software (BD Biosciences). A minimum of 20,000 events were recorded for each sample. Fluorescence minus one control (for each antibody) was used to gate the subpopulations.

Alkaline phosphatase detection was performed with alkaline phosphatase detection kit (SCR004; Millipore) according to manufacture instructions.

### Statistical Analysis

Student *t*‐test analysis was used to assess differences between control and *EDG5‐*RNAi groups. The results were considered significant if *p* < .05. Significant differences indicated using asterisks (*, *p* < .05; **, *p* < .01).

## Results

### Identification of New Genes Important for Reprogramming of Human Fibroblasts

**Figure 1:**
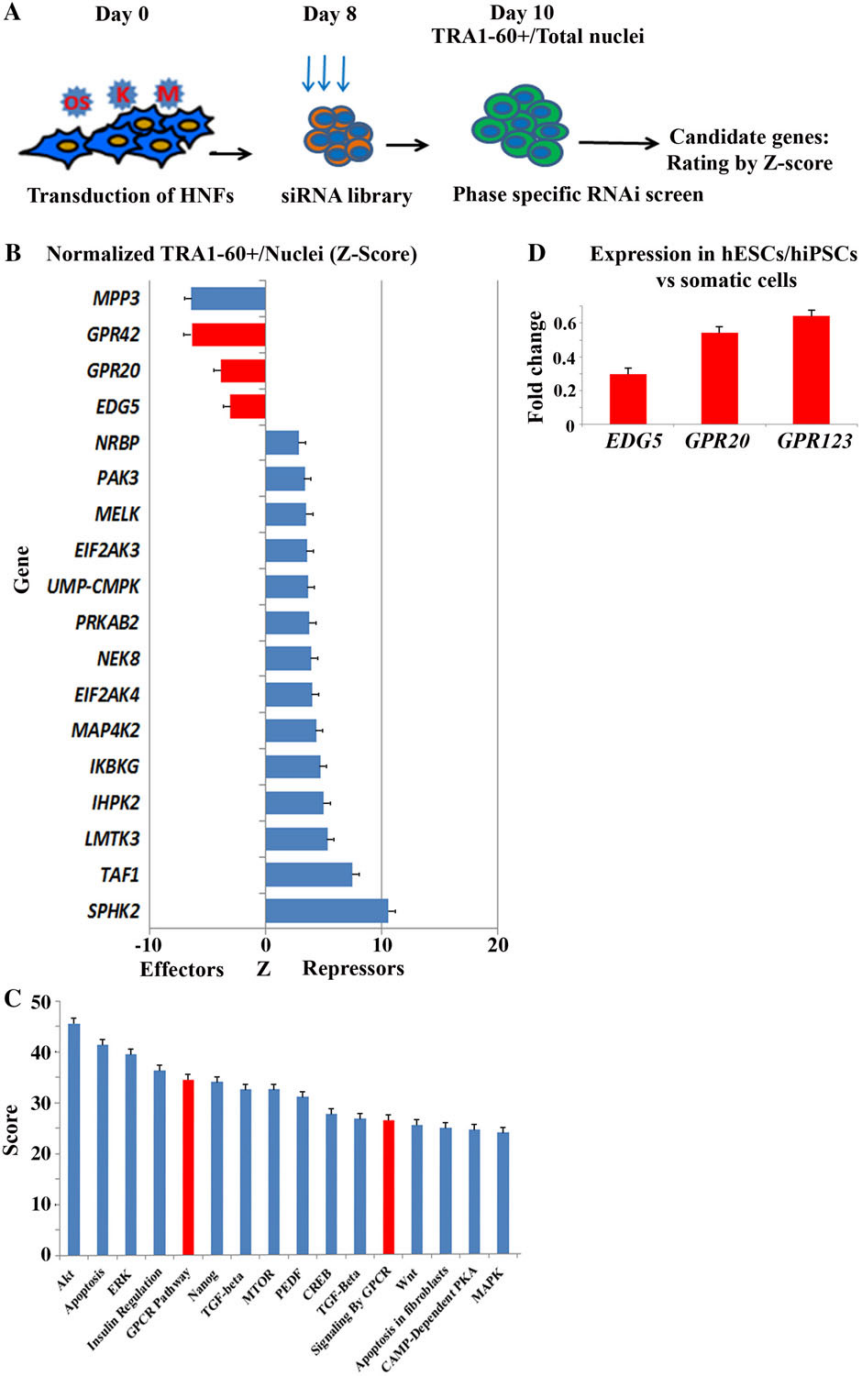
High‐throughput RNAi screen for regulators of somatic cell reprogramming. **(A):** Graphical flow of the experimental design. HNFs were transduced with *OCT4, SOX2, KLF4,* and *c‐*MYC (OSKM) and on the day 8 of reprogramming RNAi to 784 genes were applied to cells transferred to 384 well‐plate format. Colonies were examined at day10 via automated cells imaging for the expression of pluripotency cell surface marker TRA1‐60 (green) at day 10. All nuclei were stained with 4′,6‐diamidino‐2‐phenylindole. Total area for green colonies (TRA1‐60+) over the total number of nuclei was used for statistical analysis: average *Z*‐score was applied with cutoff of ±1.65 with error at 0.005 to reveal candidate genes; **(B):** *Z*‐score‐ranked distribution plot for the RNAi screen at day 10 post OSKM transduction**.** Top candidates are shown here, and the full list supplied in Supporting Information Figure S1 **.** The top three new effectors belonging to the GPCRs family *GPR42, GPR20*, and *EDG5* are shown in red; **(C**)**:** Graphical representation of the gene ontology‐enriched signaling pathway analysis of 90 candidate genes; **(D):** graphical representation of the overexpression of *EDG5, GPR20*, and *GPR123* in pluripotent cells over 100 of human somatic cells analyzed from publically available data 32. Abbreviations: GPCR, G protein‐coupled receptor; hESC, human embryonic stem cell; hiPSC, human‐induced pluripotent stem cell; HNF, Human neonatal fibroblast; MAPK, mitogen‐activated protein kinase; PEDF, Pigment Epithelium Derived Factor; PKA, protein kinase‐A; RNAi, RNA interference; siRNA, small interfering RNA; TGF, transforming growth factor.

To identify new candidate genes involved in hiPSCs generation, we developed a high‐throughput assay adapted to a 384‐well format for genome‐wide siRNA screening using the Dharmacon library (Fig. 1A). We performed siRNA‐mediated knockdown of 784 members of the different kinases and phosphatases family members within a specific phase: days 8–10 of reprogramming. We choose this period of reprogramming because at this time many cellular events (such mesenchymal to epithelial transition, increased cell proliferation, changes in cellular metabolism, and cytoskeleton remodeling) which are important for hiPSC generation take place 17, 18. Cells were examined via automated cell imaging for the expression of the cell surface pluripotency marker TRA‐1‐60 at day10 (Fig. 1A**)**. The average Z scores from three biological repeats were calculated for TRA‐1‐60+ cells versus total nuclei and a cutoff of ±1.65 was applied. This analysis resulted in identification of 68 candidate repressors and 22 candidate effectors, which included genes reported to play an important role in hiPSC generation, including *MPP3, TGFBR2, ROCK1, ATM, BUB1*, and *TESK1* (Supporting Information Fig. S1A**)** 11, 15, 27, 28, 29. Gene ontology‐enrichment pathway analysis of these candidate repressors and effectors showed enrichment of multiple biological processes and pathways, which included known (e.g., AKT, ERK, and apoptosis) and novel regulators (GPCRs) of reprogramming (Fig. 1B, 1C). Importantly, we identified six genes belonging to the GPCR family, namely *EDG5, GPR42, GPR20, GPR123, GPR116*, and *GPR37L1* as candidate effectors of the reprogramming process (Fig. 1B; Supporting Information Fig. S1A).

Our examination of the expression of the named GPCR candidates in hESC and hiPSC compared to somatic cells using publicly available microarray expression data 32 indicated that out of the six genes of interest, three, namely *EDG5, GPR20*, and *GPR123* were significantly upregulated in hESCs and hiPSCs versus somatic cells, suggesting a putative role for pluripotency acquisition and maintenance (Fig. 1D). Thus, to get new insights into their function during somatic cell‐induced reprogramming, we focused our attention on *EDG5*, also known as S1P receptor 2 (*S1P2*).

### Downregulation of *EDG5* Abrogates hiPSCs Generation

**Figure 2:**
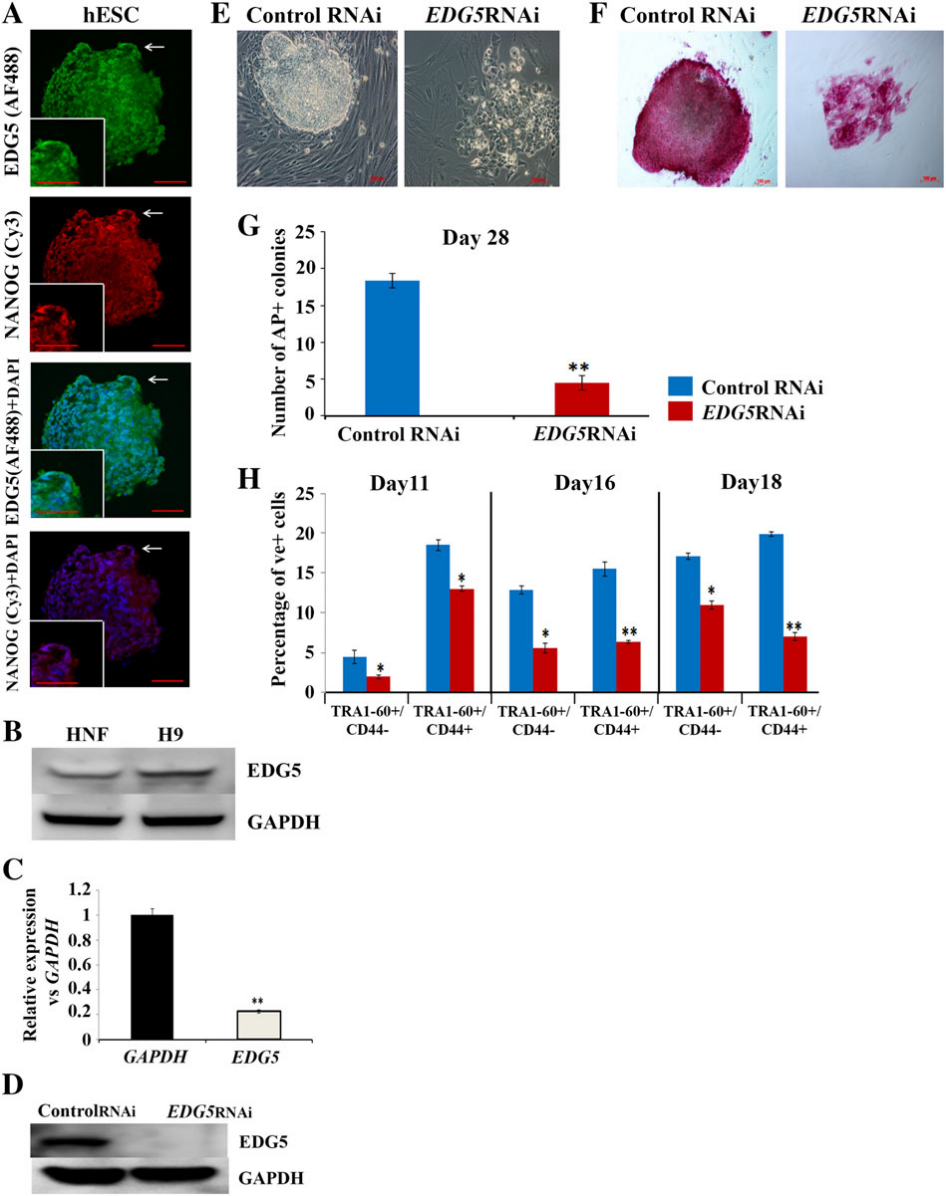
Downregulation of *EDG5* abrogates human‐induced pluripotent stem cells generation. (**A**)**:** Immunofluorescence observation of the EDG5 expression in hESCs. Scale bar 100 μm. White arrows point to the area of interest, which is shown at higher magnification in the inset. Representative examples of at least three independent experiments are shown; **(B**)**:** Representative Western blot analyses of the EDG5 expression in HNF and hESCs (H9), *n* = 3; (**C**): Real‐time quantitative polymerase chain reaction analysis of the relative expression of *EDG5* versus *GAPDH* under *EDG5‐*RNAi in hESCs. Data are shown as mean ± SEM, *n* = 3, with significance difference indicated with asterisks (**, *p* < .01); (**D**)**:** Representative Western blot analysis of EDG5 in hESCs transfected with control and EDG5siRNA, *n* = 3. GAPDH serve as a loading control. (**E, F**)**:** Representative images depicting typical colonies morphology at brightfield (**E)** and AP+ staining for control and *EDG5siRNA‐*treated colonies (**F**) at day 28 of reprogramming, *n* = 3; **(G):** graphical representation of the number of the AP+ colonies at day 28 of reprogramming upon EDG5 knockdown at days 8–10 of reprogramming process. Data are represented as mean ± SEM, *n* = 3 (**, *p* < .01); **(H)**: flow cytometry analysis of different subpopulations during the time course (at day 11, day 16, and day18) of reprogramming in control and *EDG5*siRNA‐treated groups. Data are represented as mean ± SEM, *n* = 3, with significant differences indicated using asterisks (*, *p* < .05; **, *p* < .01). Abbreviations: AP, alkaline‐phosphatase; DAPI, 4′,6‐diamidino‐2‐phenylindole; hESC, human embryonic stem cell; HNF, human neonatal fibroblast; GAPDH, glyceraldehyde‐3‐phosphate dehydrogenase; RNAi, RNA interference.

Immunofluorescence analysis demonstrated expression of EDG5 on the surface, cytoplasm, and nucleus of hESCs **(**Fig. 2A). Western blot analysis revealed higher expression of EDG5 in hESCs versus HNFs (Fig. 2B). To validate the results of high‐throughput screening, we downregulated the expression of *EDG5* by RNAi at day 8 of reprogramming (Fig. 2C, 2D**)** and assessed the number of alkaline phosphatase positive colonies at day 28. This analysis indicated a significant reduction in the number of alkaline phosphatase of pluripotent stem cell colonies (Fig. 2E–2G), thus confirming the data of the high‐throughput RNAi screen and suggesting that the GPCR family member, EDG5, may have an important role during the initiation stage of reprogramming process. To address which subpopulations were affected by *EDG5* knockdown, we performed flow cytometric analysis (Fig. 2H) which indicated a significant reduction in the percentage of fully and partially reprogrammed cells represented by the TRA1‐60+/CD44‐ and TRA1‐60+/CD44+ subpopulations, respectively, thus suggesting that the impacts of *EDG5*RNAi at the early stages of reprogramming were irreversible.

### Downregulation of *EDG5* Changes Colony Morphology and Induces Differentiation in hESCs

**Figure 3:**
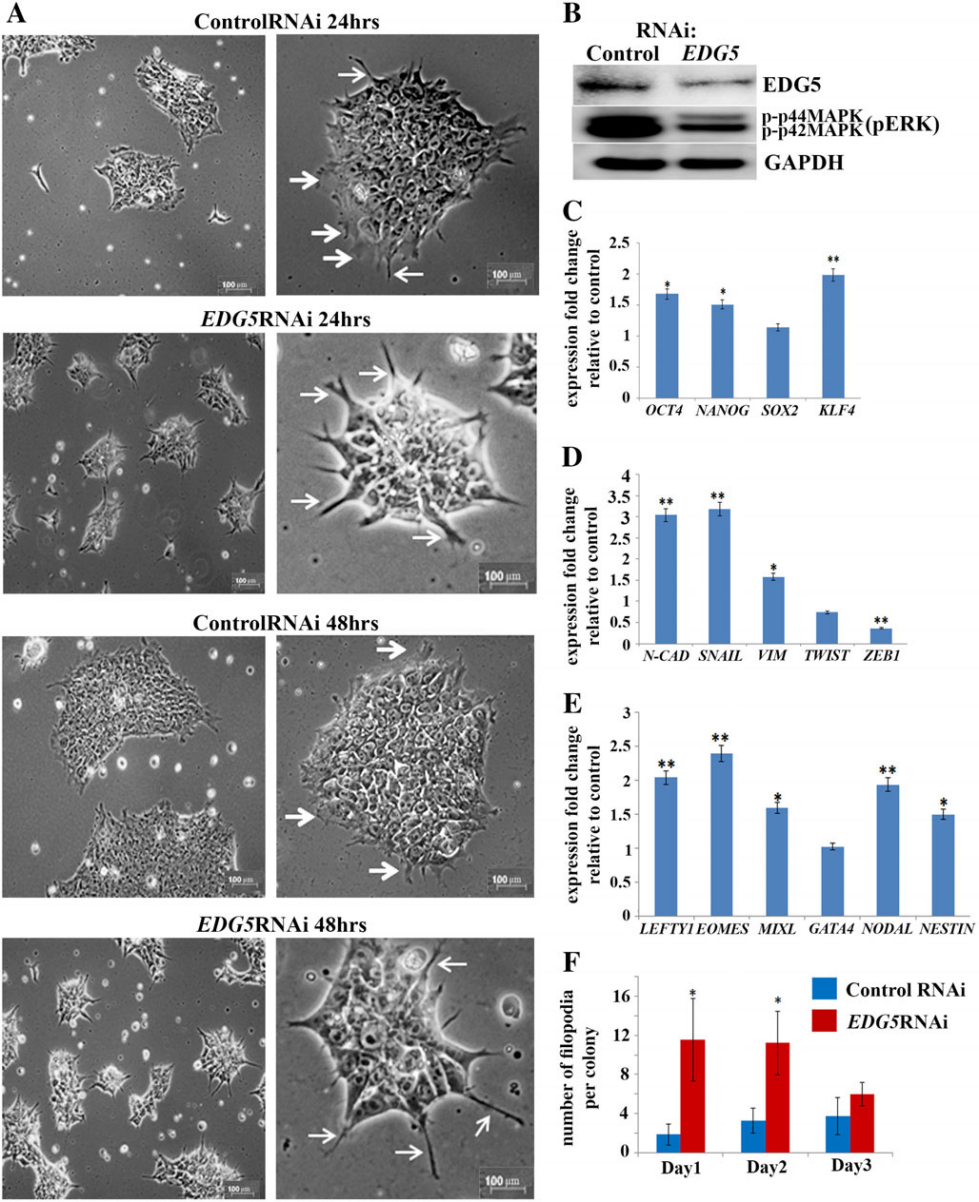
Downregulation of *EDG5* results in changes in human embryonic stem cells (hESCs) colony morphology and increased expression of differentiation marker genes. **(A):** Representative brightfield images showing hESCs colony morphology at 24 and 48 hours of transfection with control and EDG5 small interfering RNAs (siRNAs). Thick white arrows point to lamellipodia, thin arrows‐like to the filopodia‐like projections. Scale bar 100 μm, *n* = 3; **(B):** Representative Western blot analysis of the EDG5 and pERK expression in control and *EDG5‐*RNAi hESCs groups. Glyceraldehyde‐3‐phosphate dehydrogenase serve as a loading control, *n* = 3; (**C–E**): Real‐time quantitative polymerase chain reaction analysis of *OCT4*, *NANOG*, *SOX2*, and *KLF4* pluripotency marker (**C**) of *E‐cadherin*, *N*‐c*adherin*, *SAIL*, *VIMENTIN*, *TWIST*, and *ZEB1* markers (**D**) and mesendodermal markers *LEFTY1, EOMES*, *MIXL*, *GATA4*, *NODAL*, and *NESTIN* in **(E**), data presented as mean ± SEM, *n* = 3 (*, *p* < .05; **, *p* < .01); **(F):** Graphical assessment of the number of filopodia per colony in control and *EDG5siRNA‐*treated hESCs colonies at 3 days post‐transfection. **(C–F):** Data presented as mean ± SEM, *n* = 3;*, *p* < .05. Abbreviation: RNAi, RNA interference.

To address the role of EDG5 in hESC, we performed RNAi (Fig. 3A**)**. Downregulation of EDG5 was confirmed using Western blotting (Fig. 3B). Downregulation of pERK was also observed, suggesting that similarly to other cell types (hepatoma cells, CHO‐K1, and glioma cells) EDG5 in hESCs may transduce its signal via G12/13 and Gi subunits, which signal and participate in pathway leading to activation of the MAPK protein ERK 36, 38. Morphological assessment revealed the presence of long filopodia‐like projections and larger gaps between cells within the colony (Fig. 3A). As colony integrity and morphology is one of the characteristic features of the pluripotency, we examined pluripotency and differentiation markers expression. Quantitative reverse transcription polymerase chain reaction revealed increased expression of *OCT4, NANOG*, and *KLF4* (Fig. 3C), in support of the observation that some cells in *EDG5‐*RNAi colonies are positive for alkaline phosphatase staining (Fig. 2F, 2G). At the same time, *EDG5* knockdown induced a significant upregulation of *N‐cadherin* and *SNAIL* (Fig. 3D). Unlike E‐cadherin, *N‐cadherin* is not expressed in hESCs; instead, it is rapidly upregulated in both human and mouse ESC upon start of differentiation in a process akin to epithelial‐to‐mesenchymal transition (EMT) event 39. Thus, *N*‐*cadherin* and *SNAIL* upregulation in *EDG5* knockdown cells may mark the start of EMT transition instead of mesenchymal‐to‐epithelial transition (MET), which is critical for iPSCs generation. Further analysis of the genes involved in mesendodermal differentiation indicated overexpression of these markers (Fig. 3E), further supporting the start of differentiation process.

EDG5 in hESCs has been associated with the Gi, Gq, and G12/13 signaling 36, with possible preferential activation of G12/13, important for Rho small GTPase signaling 31. Rho‐ROCK signaling itself has been shown to be important for the integrity of the stem cell colony, thus supporting stem cell maintenance 40, 41. Rho family members have been implicated in regulation of the assembly of the multimolecular focal complexes associated with actin stress fibers, lamellipodia, and filopodia formation 42. The mechanisms of lamellipodia and filopodia formation in hESCs are not well‐understood but Rho family proteins, Rac, Cdc42, and RhoG have been shown to play a central role in regulation of protrusion in other cell types 43. In addition, in G12/13‐deficient mouse embryonic fibroblasts (MEFs), G13 subunit was shown to play an important role in membrane ruffles and lamellipodia formation 44. Our analysis indicated a significant reduction in lamellipodia and a dramatic increase of filopodia‐like protrusions at 24 and 48 hours after *EDG5* knockdown (Fig. 3F), corroborating data for G12/13‐deficient MEFs 44. Together, our data suggest that *EDG5* knockdown leads to upregulation of differentiation markers and an increase in filopodia formation, which will be explored further in the following results section.

### *EDG5* Downregulation Abrogates RhoA‐ROCK Signaling and Results in Cytoskeleton Dysregulation in hESCs

It is well‐established that G12/13‐coupled GPCRs signaling leads to the activation of RhoA, which plays a central role in the organization of the actin cytoskeleton through its ability to stimulate the formation of actomyosin‐based structures 45, regulation of microtubule dynamics, stress fiber formation, and transcriptional activity 43. This led us to examine the possible effects of *EDG5* downregulation on the EDG5‐G12/13‐RhoA‐ROCK axis.

**Figure 4:**
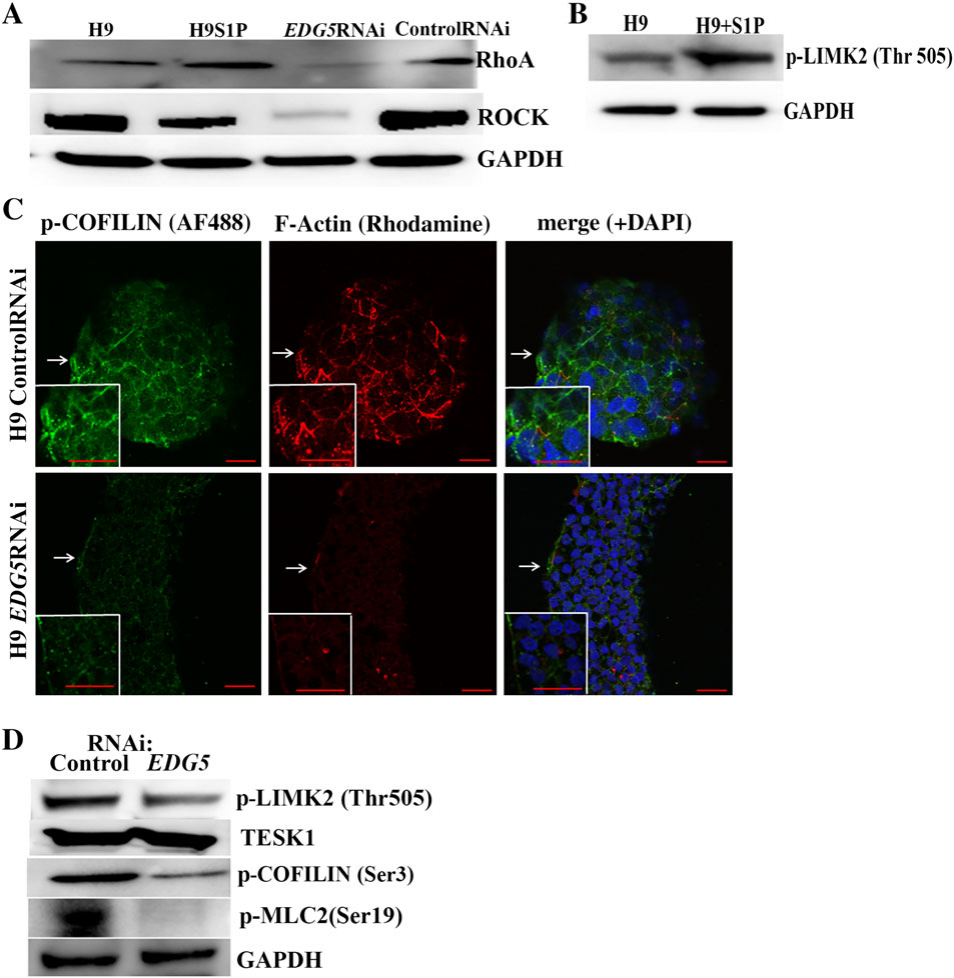
*EDG5* downregulation abrogates RhoA‐ROCK signaling and induces alteration in COFILIN/F‐actin cytoskeleton organization in human embryonic stem cells (hESCs). **(A):** Representative Western blot analysis of RhoA and ROCK expression in hESCs (H9), hESCs stimulated with 20 μM S1P for 90 minutes (H9S1P), *EDG5‐*RNAi hESCs, and control RNAi hESCs. GAPDH used as a loading control, *n* = 3; **(B):** representative Western blot analysis of p‐LIMK2 (Thr505) in hESCs (H9) and hESCs stimulated with 20 μM S1P for 90 minutes. GAPDH‐loading control, *n* = 3; **(C):** representative immunofluorescence images of hESCs colonies treated with *EDG5* and controls small interfering RNAs (siRNAs) for 72 hours. White arrows point to the area of interest which is shown at higher magnification in the inset. Scale bar = 100 μm, *n* = 3; **(D):** representative Western blot of p‐LIMK2 (Thr505), TESK1, p‐COFILIN (Ser3), and p‐MLC2 (Ser19) expression in hESCs colonies after 72 hours from *EDG5* and controls siRNAs, *n* = 3. Abbreviations: GAPDH, glyceraldehyde‐3‐phosphate dehydrogenase; RNAi, RNA interference; S1P, sphingosine‐1‐phosphate.

Western blot analysis indicated downregulation of RhoA and ROCK upon knockdown of *EDG5* in hESCs, whereas stimulation of EDG5 by S1P had the opposite effect on RhoA expression (Fig. 4A; Supporting Information Fig. S2A**‐** S2A’). Stimulation of EDG5 by S1P also induced an increase in p‐LIMK2 (Thr505) form, suggesting an upstream role for EDG5 in COFILIN phosphorylation (Fig. 4B; Supporting Information Fig. S2B). It is known that active ROCK phosphorylates LIMK2 at Thr505 (but not LIMK1), increasing its kinase activity toward COFILIN 46. The COFILIN/actin depolymerizing factor family of proteins plays a critical role in actin depolymerization, which is essential for recycling actin subunits to support new filament growth. As we detected reduced protein expression of RhoA and ROCK under *EDG5‐*RNAi, we performed immunocytochemistry, which revealed a significant loss of prominent staining for p‐COFILIN(Ser3) and filamentous actin under *EDG5‐*RNAi conditions in hESCs (Fig. 4C). These observations were supported by Western blotting analysis, which demonstrated reduction in expression of p‐LIMK2 (Thr505) and p‐COFILIN (Ser3) (Fig. 4D; Supporting Information Fig. S2C). In addition to LIMK2, TESK1 is another regulator of COFILIN, as both have been shown to phosphorylate and inactivate COFILIN at Ser3, thus behaving as an actin severing factor 27. Western blot analysis did not detect changes in TESK1 expression (Fig. 4D; Supporting Information Fig. S2C), suggesting that TESK1 is not a downstream effector of the RhoA‐ROCK signaling in our experimental settings 47. Work performed in other cell types (Chinese hamster ovary (CHO) cells and fibroblasts 48) has shown that activation of the EDG5 results in RhoA‐dependent increase in myosin light chain (MLC) phosphorylation and prominent stress fiber formation. Nonmuscle MLC2 has been described as a key substrate for ROCK signaling in hESCs 40. Given the downregulation of Rho and ROCK upon *EDG5* knockdown, we investigated the expression of phosphorylated form of MLC2, which was also downregulated as demonstrated by Western blot analysis (Fig. 4D; Supporting Information Fig. S2C). In conclusion, these data indicate that both consecutive pathways were important for actin cytoskeleton organization, namely Rho‐ROCK‐LIMK2‐p‐COFILIN and Rho‐ROCK‐p‐MLC2 were compromised under *EDG5‐*RNAi in hESCs.

### Downregulation of *EDG5* Disrupts PAXILLIN Cytoskeleton and FA in hESCs

**Figure 5:**
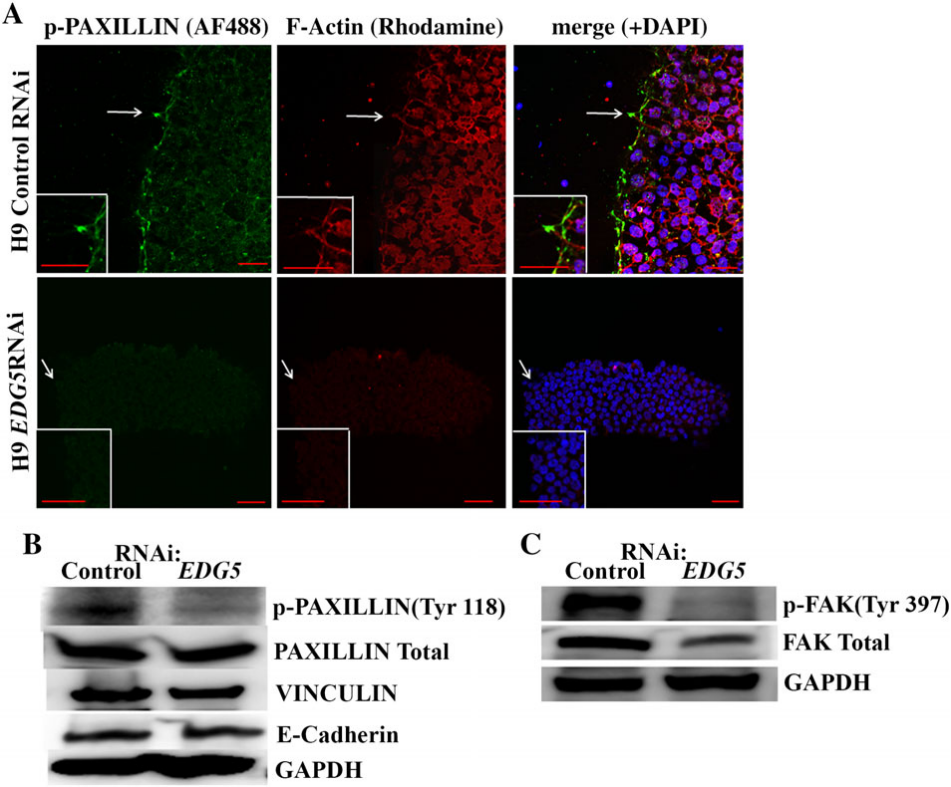
PAXILLIN cytoskeleton and focal adhesion are altered upon downregulation of *EDG5*. **(A):** Representative immunofluorescence staining for p‐PAXILLIN (Tyr118) and F‐actin in control and *EDG5* small interfering RNA (siRNA)‐treated human embryonic stem cells (hESCs). White arrows point to the area of interest, which is shown at higher magnification in the inset. *n* = 3. Scale bar 100 μm. **(B, C):** Representative Western blot analysis of p‐PAXILLIN (Tyr118), PAXILLIN Total, VINCULIN, and E‐cadherin in control and *EDG5*siRNA‐treated hESCs. GAPDH used as a loading control, *n* = 3. Abbreviations: DAPI, 4′,6‐diamidino‐2‐phenylindole; GAPDH, glyceraldehyde‐3‐phosphate dehydrogenase; RNAi, RNA interference.

Another component of the hESCs cytoskeleton shown to be important for colony morphology is PAXILLIN 49. PAXILLIN is a multidomain protein that localizes primary to sites of cell adhesion to extracellular matrix, called FAs. hESCs and hiPSCs display large, PAXILLIN‐positive FA at the edge of the colonies. A strong contractile actin fence and large adhesions have been shown to be important for maintaining hESCs colony morphology 50. We examined the effect of the *EDG5* knockdown on PAXILLIN cytoskeleton of hESCs (Fig. 5A, 5B**)**. Our results indicate that p‐PAXILLIN (Tyr118) is lost from FAs sites upon *EDG5‐*RNAi conditions (Fig. 5A, 5B; Supporting Information Fig. S3A**)**: the same was found under ROCK inhibition **(**data not shown). FAK is a substrate for ROCK; in accordance, the expression of p‐FAK (Tyr397) and the total FAK were significantly reduced upon EDG5 knockdown in hESCs (Fig. 5C; Supporting Information Fig. S3B), thus suggesting that FA is also affected in hESC (in addition to the COFILIN‐PAXILLIN‐F‐actin cytoskeleton) as result of *EDG5* downregulation.

### Downregulation of *EDG5* Induces Alteration in the Cytoskeleton and Loss of FAs at hiPSCs During Somatic Reprogramming

**Figure 6:**
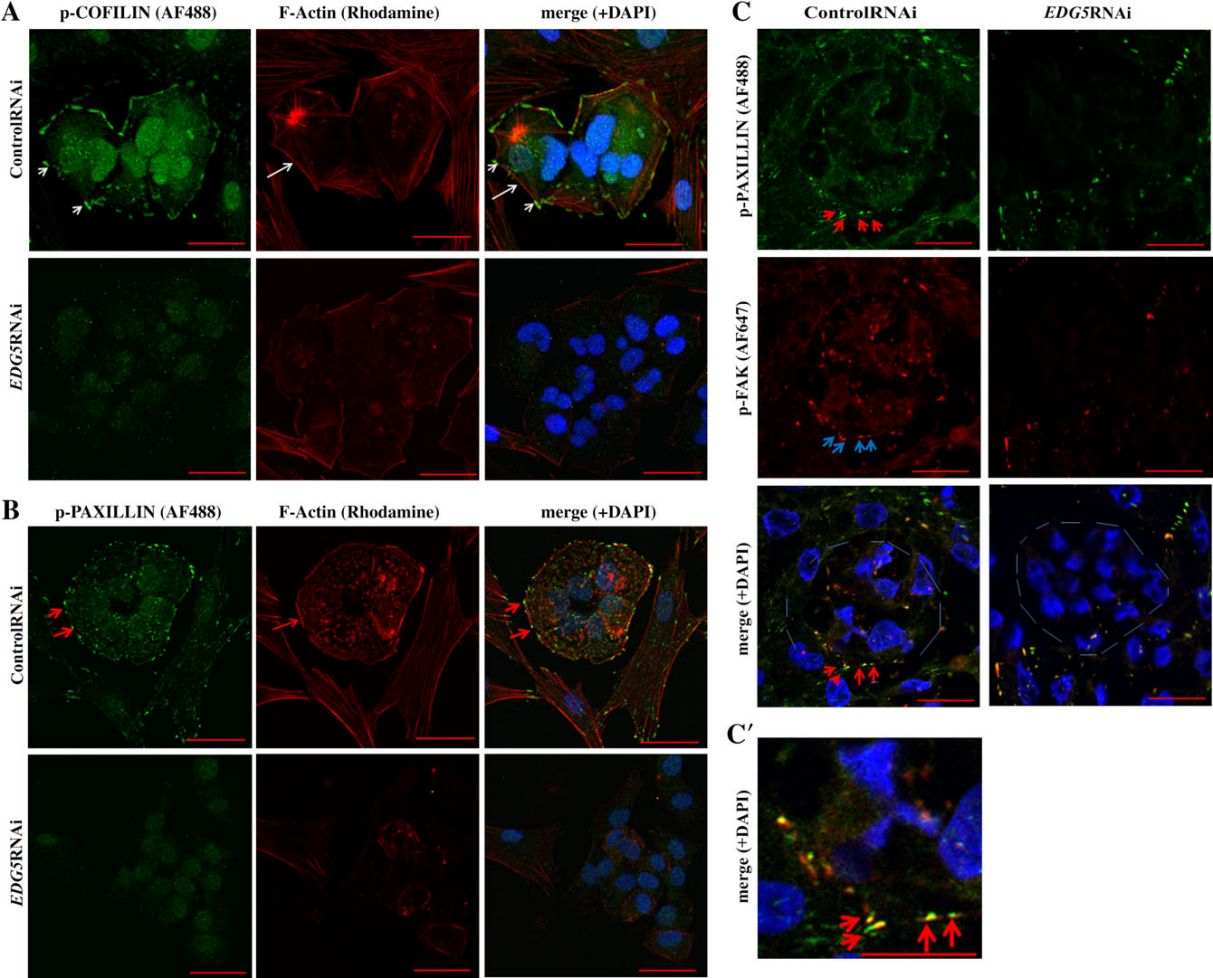
Downregulation of *EDG5* during reprogramming effect COFILIN/PAXILLIN/F‐actin organization in newly formed colonies. **(A‐C):** Typical confocal fluorescence images showing p‐COFILIN (Ser3), p‐PAXILLIN (Tyr118), p‐FAK (Tyr397), and F‐actin stained as indicated in colonies emerging at day 10 of *OCT4, SOX2, KLF4*, and *c‐MYC* transduction and treated with control or *EDG5* small interfering RNA (siRNA) from day 8 to day10 of reprogramming, *n* = 3**. (A):** Short white arrows point to localization of p‐COFILIN (Ser3). Long white arrow pointed to F‐actin thick fibers. Note that a thick bundle of F‐actin surrounds the colony. **(B):** Short red arrows point to p‐PAXILLIN; long red arrow points to the F‐actin. Note at the merge images, colocalization of p‐COFILIN **(A)** and p‐PAXILLIN **(B)** with F‐actin at the end of F‐actin fibers. None of such structures can be observed in *EDG5*siRNA‐treated groups. Representative images from at least three independent biological replicates are shown. **(C–C′):** Red arrows pointed to p‐PAXILLIN (Tyr118) with the focus plane for focal adhesions sites at control colonies; blue arrows pointed for p‐FAK (Tyr397) at the sites of focal adhesions and at the merged images; red arrows pointed for colocalization of p‐PAXILLIN and p‐FAK at the control colonies, while no such pattern of expression can be seen at *EDG5‐*RNAi colonies. Please note that in EDG5‐RNAi samples, some cells express p‐PAXILLIN and p‐FAK, but without expression or colocalization at the edges of the colonies, circled by the thin light blue lines. **(C′):** Magnification of ×4 of the merge images showing (red arrows) colocalization of p‐PAXILLIN (green) and p‐FAK (red) in control colonies. Abbreviations: AP, alkaline‐phosphatase; DAPI, 4′,6‐diamidino‐2‐phenylindole; RNAi, RNA interference.

In hiPSCs, FAs act as a functional signaling platform for p‐FAK (Tyr 397) and p‐PAXILLIN (Tyr118) and a “cornerstone” for the successful generation of hiPSCs colonies 50. In agreement with our observation in hESCs, immunofluorescence analysis of the p‐COFILIN‐F‐actin and p‐PAXILLIN–F‐actin organization at the hiPSCs colonies at day 10 post‐transduction demonstrated the presence of a ring‐like structure around the hiPSC colonies, which showed the expression of p‐COFILIN, p‐PAXILLIN, and F‐actin (Fig. 6A). Knockdown of *EDG5* from days 8 to 10 of reprogramming abolished the presence of p‐COFILIN and p‐PAXILLIN and disrupted the filamentous actin structures at the edge of the colonies (Fig. 6A, 6B), leading to disperse spreading of the cells within the newly emerging colonies and development of filopodia‐like protrusions (black arrows, Supporting Information Fig. S5), akin to morphological features we observed in hESC upon *EDG5* downregulation (Fig. 3A**)**. In contrast to the control colonies, colocalization of p‐FAK (Tyr397) with p‐PAXILLIN (Tyr118) was not observed at the edges of the *EDG5‐*RNAi colonies at day 10 of reprogramming (Fig. 6C, 6C’), corroborating previous data from 50 and indicating an important role for EDG5 signaling in hiPSCs development. Surprisingly, we did not find a difference in the expression of another important component of FAs: VINCULIN. Immunofluorescence analysis of VINCULIN distribution during reprogramming did not reveal differences between the control and *EDG5* inhibited groups (Supporting Information Fig. S4), corroborating out data on hESCs (Fig. 5B; Supporting Information Fig. S3A**)**. This may be explained by the fact that localization of VINCULIN is regulated by TESK1 via integrin signaling cascade 47; however, TESK1 signaling is unaffected by downregulation of *EDG5* as shown in Figure 4D and Supporting Information Figure S2C. Also, in agreement with data on hESCs about unchanged expression of E‐cadherin (Fig. 5B; Supporting Information Fig. S3A) in response to *EDG5* knockdown, we did not detect alteration on E‐cadherin expression by immunofluorescence during reprogramming under *EDG5*‐RNAi conditions **(**Supporting Information Fig. S4). In summary, our data suggest that downregulation of EDG5 during the initiation stage of reprogramming results in ablation of FAs at the edges of the colonies mediated by the significant reduction of p‐PAXILLIN/F‐actin and FAK.

### The Role of FAK Signaling During Somatic Cell‐Induced Reprogramming

**Figure 7:**
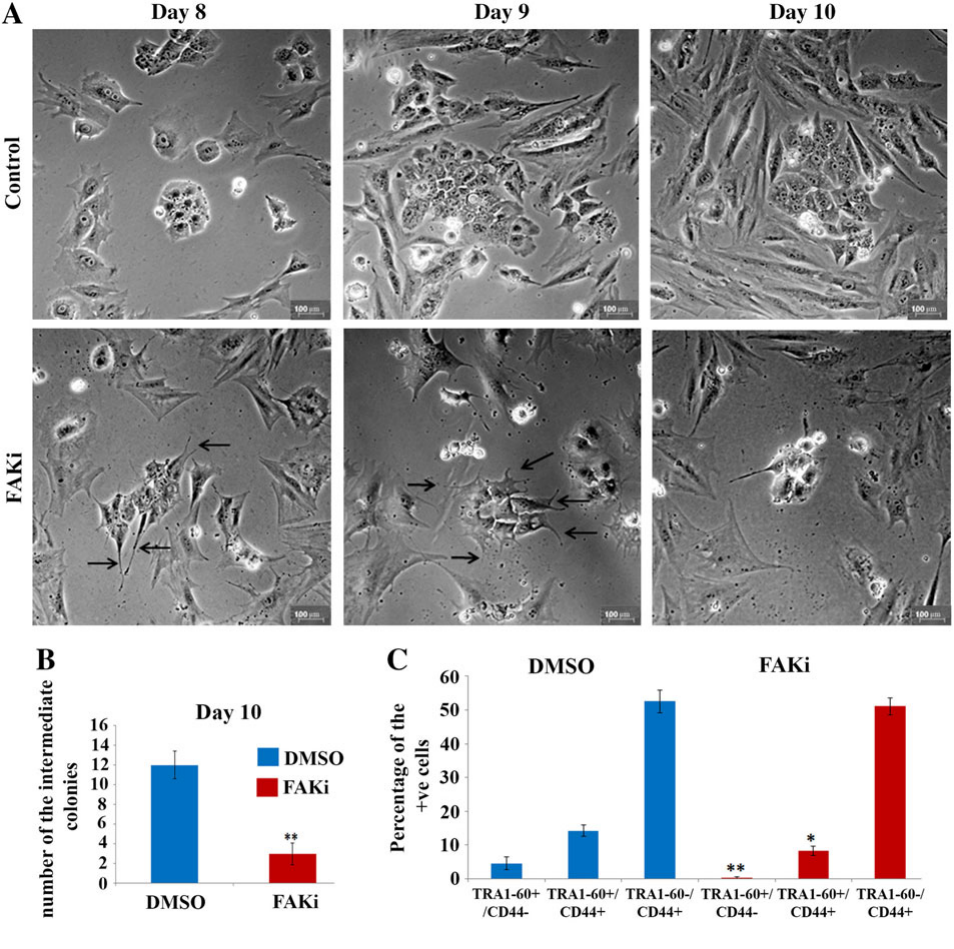
FAK inhibition abrogates colonies development during the reprogramming process. (**A**)**:** Brightfield representative images of the typical colonies morphology developed in control (DMSO) and FAK inhibitor (2 μM, PF562271; FAKi) groups during the time course of FAKi treatment, from day 8 till day 10, *n* = 3; **(B):** graphical representation of the number of the intermediate colonies at day 10 in control Dimethyl sulfoxide (DMSO) and FAK inhibitor group (FAKi). Data are mean ± SEM; **, *p* ≤ .001; *n* = 3. **(C):** Flow cytometric analysis of TRA1‐60+/CD44‐, TRA1‐60+/CD44+, and TRA1‐60‐/CD44+ subpopulations at day 10. FAK inhibitor treatment started at day 8. Data are mean ± ; *n* = 3. *, *p* < .05; **, *p* < .01.

Given the downregulation of p‐FAK (Tyr397) and loss of colony integrity upon *EDG5‐*RNAi (Fig. 5C, 6C), we went on to investigate the impact of FAK inhibition using a specific inhibitor (2 μM, PF562271; FAKi) from day 8 till day 10 of reprogramming (to correspond with the same window when RNAi screen was performed; Fig. 1A). Morphological examination of the colonies developed under FAKi conditions and *EDG5* knockdown revealed several similarities, namely the development of protrusions, sparse distribution of the cells, and loss of clear colony edges (Fig. 7A; Supporting Information Fig. S5), further supporting our data of the importance of EDG5‐FAK regulation for hESCs/hiPSCs. Comparison of the number of emerging intermediate colonies at day 10 demonstrated a significant reduction in the FAK‐inhibited group (Fig. 7B). Flow cytometric analysis revealed that the percentage of the partially and fully reprogrammed subpopulations was significantly reduced upon FAK inhibition (Fig. 7C), suggesting a link between EDG5 signaling and FAK and corroborating previous data about the important role for FAK in reprogramming process 50.

## Discussion

Since the discovery that somatic cells could be reprogrammed to iPSCs in 2006 by Yamanaka and colleagues 51, vast amount of research has be performed to try to improve the reprogramming efficiency and to better understand the molecular machinery of pluripotency acquisition and maintenance. Undoubtedly, numerous studies which reported identification of a new effectors or barriers of reprogramming process have broaden our knowledge about complex interaction of the different signaling pathway and reprogramming mechanisms 11, 19, 52 and have highlighted an important role for MET, metabolism, apoptosis, cytoskeleton rearrangement, autophagy, immune response, cell cycle alterations, epigenetics, and many others for the effective hiPSCs generation.

In our research, we used a 384‐well plate format for small interfering RNA (RNAi) screening of the library of 784 different kinases and phosphatases. This screening strategy allowed us to identify in addition to the known players in reprogramming new candidate effectors and repressors of this process. This screen revealed enrichment in GPCRs signaling and identified six new effectors (GPR42, GPR20, EDG5, GPR123, GPR116, and GPR37L1) belonging to this family. We focused our attention on EDG5 and showed that downregulation of *EDG5* resulted in loss of typical colony morphology, acquisition of filopodia, dysregulation of actin cytoskeleton, overexpression of mesendodermal and EMT markers, and a significant reduction in the number of alkaline‐positive colonies in hESCs. Similarly, downregulation of *EDG5* during the initiation stages of human somatic cell‐induced reprogramming led to similar dysregulation of cytoskeleton, colony integrity, FAs, and reduced number of the pluripotent stem cell colonies at the end of the reprogramming process. Accordingly, these data imply that in hESCs, *EDG5* functions cannot be substituted by its close EDG family member, EDG3, which has been suggested to compensate for Edg5 null mice, which are viable, fertile, and developed normally 53. Corroborating our data, it has been reported that MEFs null for EDG5 show a significant reduction in Rho activation but no effect on PLC activation, calcium mobilization, or adenylyl cyclase inhibition, suggesting preferential signaling via EDG5 to G12/13 α subunit, important for Rho‐ROCK signaling 53. At the same time, infection of MEFs with constitutively active G12 together with reprogramming factors led to a significant reduction in alkaline phosphatase colonies 15, suggesting that affecting the same signaling axis via one effector on the same signaling network may lead to opposite effects in different experimental condition and pointing to the complexity of the reprograming process as well as differences in human versus mouse cells.

In hESCs, S1P receptors are linked to different G proteins, allowing this signaling pathway to elicit a variety of specific responses through the activation of Gi, Gq, G12/13, and Gs which control survival, proliferation, migration, and self‐renewal 31, 35. Our experiments suggest that EDG1 and EDG3 cannot compensate for the ablation of EDG5 signaling. The function of EDG1 and EDG3 in hESCs is not well‐known; thus, additional studies are needed to understand the S1P‐mediated effects on reprogramming and whether culture media supplementation with S1P rescues EDG5 downregulation. In addition, it will be of interest to investigate whether supplementation of the culture media with S1P (which in hESCs can signal via EDG1, EDG3, and EDG5 receptors) can be beneficial for reprogramming.

Regardless the fact that G protein signaling is recognized as a key functional regulator of hiPSCs generation 11, this is the first study dedicated to the functional role of the EDG family GPCRs member, *EDG5*, in this process. As EDG5 signaling can be mediated via three different G‐α subunits resulting in the different cellular outcomes, we focused our study to the EDG5‐G12/13‐RoA‐ROCK signaling pathway. Our study demonstrated that *EDG5* downregulation in hESCs abrogated Rho‐ROCK signaling and led to COFILIN/PAXILLIN/F‐actin cytoskeleton abnormalities. Earlier, the importance of actin filament organization for human somatic cell reprogramming was suggested by 11, 28, 31 and recognized as a significant hit through identification of candidate genes involved in cell motility and adhesion during hiPSCs generation 16. Importantly, it was shown that during MET, the actin cytoskeleton is reorganized from actin stress fibers to cortical actin, a process tightly regulated by phosphorylation of COFILIN on Ser3 by LIMK2 and TESK1 activity 27. In agreement with this, our data demonstrated a reduction at the active form of LIMK2 and p‐COFILIN (Ser3), which led to the ablation of the filamentous actin organization upon downregulation of *EDG5*. This also occurred during the somatic cell‐induced reprogramming, thus highlighting for the first time that the importance of the EDG5‐G12/13 signaling is the maintenance and induction of pluripotency.

It is known that actin filaments generate forces that drive changes in cell shape and mechanics through their interaction with myosin molecular motors. In fact, RhoA‐GTPase/Rho‐associated ROCK‐myosin‐II signaling can alter tension of the actin cytoskeleton and regulate survival of individual hESCs 54. Furthermore, actin‐myosin colocalization was shown to be enhanced at the edge of the colonies, and the block of myosin II activity leads to loss of colonies 55. Our analysis for the active form of myosin II, p‐MLC2 (Ser19), revealed its significant downregulation, suggesting that RhoA‐ROCK‐dependent pathway important for actin cytoskeleton organization and operating via phosphorylation of cofilin and MLC2 was severely dysregulated upon EDG5 knockdown, thus providing a potential mechanism for loss of hiPSC colonies during the reprogramming process. In previous reports, it has been reported that ROCK inhibition with Y‐27632 for 24 hours facilitates generation of hiPSCs 56. At first glance, these data may seem contradictory to our findings. However, it is well known that ROCK inhibition render hESCs less sensitive to the environmental changes (e.g., dissociation and detachment during single‐cell propagation), thus improving their plating efficiency 57, 58. We found that in contrast to the complete absence of the filamentous actin upon *EDG5* knockdown, inhibition of ROCK signaling pathway led to reduced staining for filamentous actin in hESCs but not complete ablation (data not shown), suggesting that a direct comparison is not valid.

The assembly of filamentous actin was shown to play a direct role in controlling cytoskeletal and morphological aspects of the contact guidance response in hESCs 59. Thus, it can be hypothesized that remodeling of the actin cytoskeleton during the transition from the somatic to pluripotent stem cells ultimately will have an impact on the reorganization of the all cellular systems responsible for numerous biological processes as cellular movement, adhesion, substrate interaction, gene expression, and many others. Actin cytoskeleton is widely involved in cellular movement and chemotaxis, but very little is known about regulation of these cellular functions as in hESCs/hiPSCs 60, 61 and during human somatic cell reprogramming. Also, regardless of the fact that genes involved in the actin cytoskeleton remodeling were named as hits for hiPSCs generation, our understanding of the biology and regulation of these processes during reprogramming is very naive and calls for additional studies. With regards to this, it was shown that *EDG5* expressing CHO cells exhibited inhibition of migration and chemotaxis toward Insulin–like growth factor 1(IGF1) in response to the S1P concentration gradient 48, thus indicating that S1P and EDG5’s potential action in hESCs migration and chemotaxis are additional subjects for further study, as this may be important for single‐cell clonal expansion and propagation of hiPSCs.

Another aspect of our research is the link between actin cytoskeleton and FAs during reprogramming. FAs, the cell's mechano‐transducing units, were recently reported to be important for the generation of hiPSCs 50. PAXILLIN is a multifunctional and multidomain FA adapter protein, which serves an important scaffolding role at FAs by recruiting structural and signaling molecules involved in cell movement and migration. Tyrosine 118 phosphorylation of PAXILLIN, the main residue for PAXILLIN phosphorylation by FAK, provides a scaffold for establishment of FA. Our data demonstrated that knockdown of *EDG5* resulted in loss of PAXILLIN (Thr 118) which may be important for FA‐dependent regulation of cell morphology and hiPSCs development 50. The phosphorylation of PAXILLIN by FAK has been suggested to mediate transduction from cell adhesion to the reorganization of the cytoskeleton required for cell movement 62. Application of a specific FAK inhibitor (PF562271) during the reprogramming window resulted in a significant reduction in the number of pluripotent stem cell colonies, further supporting our data obtained with *EDG5* knockdown. Furthermore, our data provide evidence that EDG5 plays an important role for the assembly of FAs as its downregulation resulted in significant decrease on the expression of two key components, namely p‐FAK and p‐PAXILLIN, in hESC and during reprogramming of human fibroblasts to hiPSC.

## Summary

Using an RNAi screen at the initiation stage of reprogramming, we identified 90 new potential effectors and repressors of reprogramming. Of the 22 effectors, six new genes belonging to the GPCR family were identified. Detailed analysis of the *EDG5* (*S1P2*) downregulation in hESC and during reprogramming to hiPSC demonstrated alteration of the typical stem cell morphology, acquisition of filopodia, which was associated with downregulation of RhoA‐ROCK signaling, reduced expression of p‐LIMK2 (Thr505), ablation of the p‐COFILIN (Ser3) expression, and a significant reduction in filamentous F‐actin. Also, our data revealed alteration at the Rho‐ROCK‐p‐MLC2 signaling, the second axis important for actin cytoskeleton organization. Additionally, in agreement with that FAK is a substrate of ROCK, the expression of p‐FAK (Tyr 397) was reduced; consequently, p‐PAXILLIN (Tyr118), important for FAs organization at the edges of the colonies, was lost. Thus, we concluded that EDG5 signaling via G12/13‐RoA‐ROCK is important for (a) proper F‐actin cytoskeleton organization in hESCs and its remodeling during generation of hiPSCs and (b) the assembly of the PAXILLIN/F‐actin‐FAK FA complexes, which are important for pluripotency and colony integrity as summarized in the Graphical Abstract (Supporting Information Fig. S6). Application of the specific FAK inhibitor (PF562271) at day 8 to day 10 of the reprogramming process significantly reduced the number of developing colonies, further supporting our data. Thus, *EDG5* is a new indispensable gene for acquisition and maintenance of pluripotency.

## Author Contributions

I.N.: conception/design, collection and/or assembly of data, data analysis and interpretation, figure preparation, manuscript writing, final approval of manuscript; L.C., P.B., and K.G.: collection and/or assembly of data, data analysis and interpretation, final approval of manuscript; L.A. and G.L.: conception/design, financial support, final approval of manuscript; M.L.: conception/design, data analysis and interpretation, manuscript writing, financial support, final approval of manuscript; A.S.: statistical analysis and data interpretation.

## Disclosure of Potential Conflicts of Interest

L.C. declared employment/leadership position with Fujifilm Diosynth Biotechnologies UK Ltd. The other authors indicated no potential conflicts of interest.

## Supporting information


**Figure S1 Z‐score ranked distribution plot of all identified candidates from the RNAi screen.** 20 genes with Z scores of <1.65 are considered potential candidate effectors of reprogramming. 68 genes, with Z scores of >1.65 are considered potential candidates that are repressors. The top 3 effectors (**GPR42, GPR20 and EDG5)** belonging to GPCRs family are shown in red bars**.** In addition, **GPR123**, **GPR116** and **GPR37L1** were identified as potential effectors of reprogramming (red bars). Genes, which were identified previously and recognized as primary hits in human cells (*MPP3, TGFBR1, BUB1B, BMPR2, AKT1, NME5, ROCK2, RPS6KB2, TESK1, BMPR2, MELK, SPHK2*) cells. Are shown in blue bars: (*MPP3, TGFBR1, BUB1B, BMPR2, AKT1, NME5, ROCK2, RPS6KB2, TESK1, BMPR2, MELK, SPHK2*) cells.

**Figure S2 Densitometry analysis of the Western Blot data presented at Figure4A‐B and D.** (A‐A’) Quantification of RhoA and ROCK protein expression; (**B**) Quantification of the p‐LIMK2 expression; (**C**) Quantification of the expression of p‐LIMK2, TESK1, p‐COFILIN and p‐MLC2. * p < .05.

**Figure S3 Densitometry analysis of the Western Blot data represented at** Figure 5B‐C**. (A)** Quantification of the expression of p‐PAXILIN, PAXILIN Total, VINCULIN and E‐cCadherin**; (B)** Quantification of the expression of p‐FAK and total FAK. * p < .05.

**Figure S4 Organization of VINCULIN cytoskeleton is not altered upon *EDG5* downregulation during day 8–10 of reprogramming process. (A)** Typical confocal immunofluorescence observation of the VINCULIN and E‐Cadherin, in Control and *EDG5* siRNA treated groups during OSKM reprogramming at Day10. Scale bar 100 μm.

**Figure S5 *EDG5* downregulation alters the typical morphology of the emerging hiPSCs colonies. (A)** Representative bright‐field images of the Control and *EDG5* siRNA treated groups during OSKM reprogramming from day 8 till day 10. Black arrows point to the numerous projections in *EDG5* siRNA treated RNAi cells. Note sparse distribution and absence of the define edges in *EDG5* siRNA treated RNAi, n = 3. Scale bar 100um.

**Supplement Table 1**: XXX.
