# Supplementary figures and images for: Endothelial Differentiation G Protein‐Coupled Receptor 5 Plays an Important Role in Induction and Maintenance of Pluripotency
Source: Stem Cells. 2019 Feb 7;37(3):318–31. doi: 10.1002/stem.2954 (PMC6446721; doi:10.1002/stem.2954)

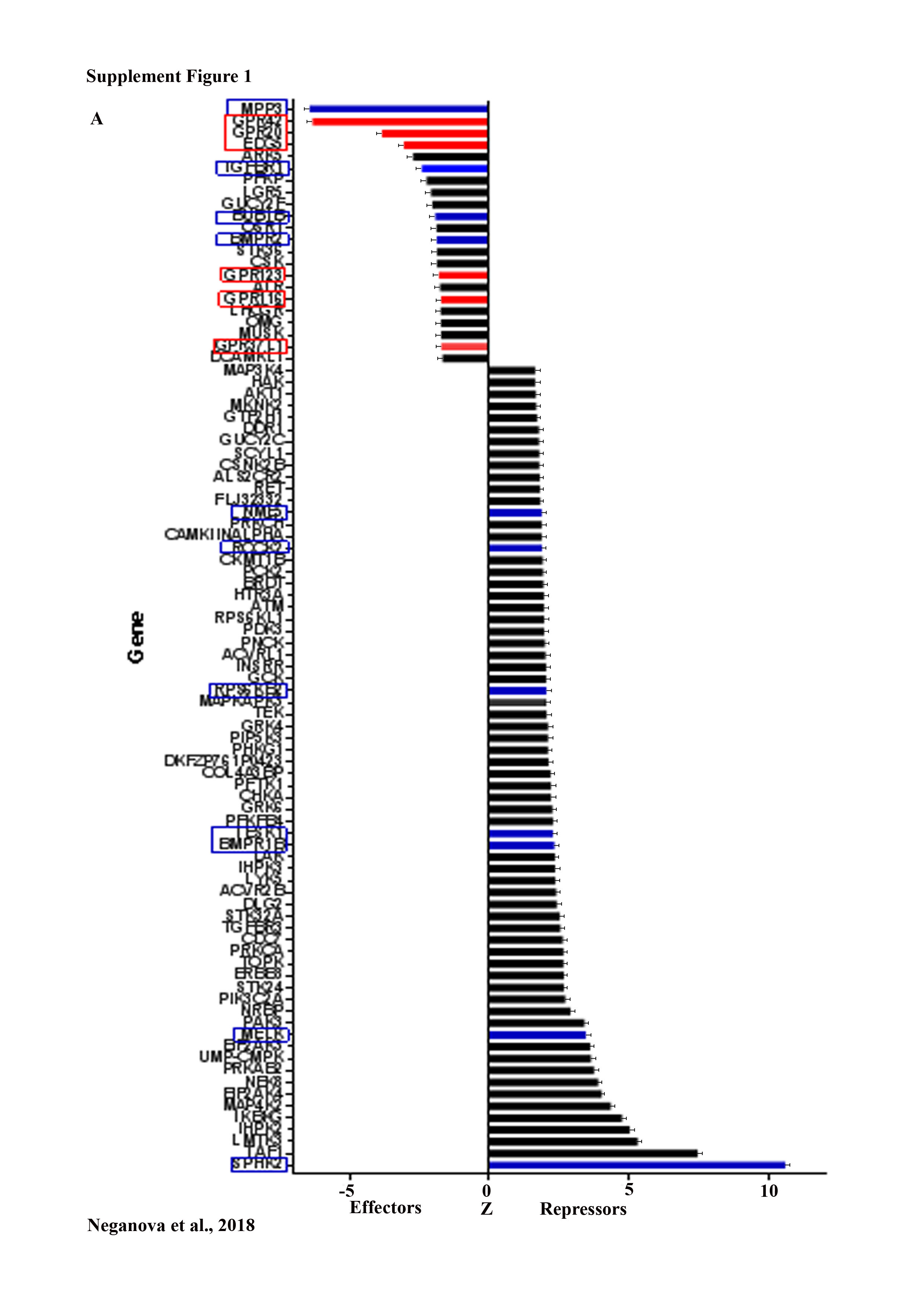

Supplement: Supplementary file 1 — Figure S1 Z‐score ranked distribution plot of all identified candidates from the RNAi screen. 20 genes with Z scores of <1.65 are considered potential candidate effectors of reprogramming. 68 genes, with Z scores of >1.65 are considered potential candidates that are repressors. The top 3 effectors (GPR42, GPR20 and EDG5) belonging to GPCRs family are shown in red bars. In addition, GPR123, GPR116 and GPR37L1 were identified as potential effectors of reprogramming (red bars). Genes, which were identified previously and recognized as primary hits in human cells (MPP3, TGFBR1, BUB1B, BMPR2, AKT1, NME5, ROCK2, RPS6KB2, TESK1, BMPR2, MELK, SPHK2) cells. Are shown in blue bars: (MPP3, TGFBR1, BUB1B, BMPR2, AKT1, NME5, ROCK2, RPS6KB2, TESK1, BMPR2, MELK, SPHK2) cells. [file STEM-37-318-s001.jpg]

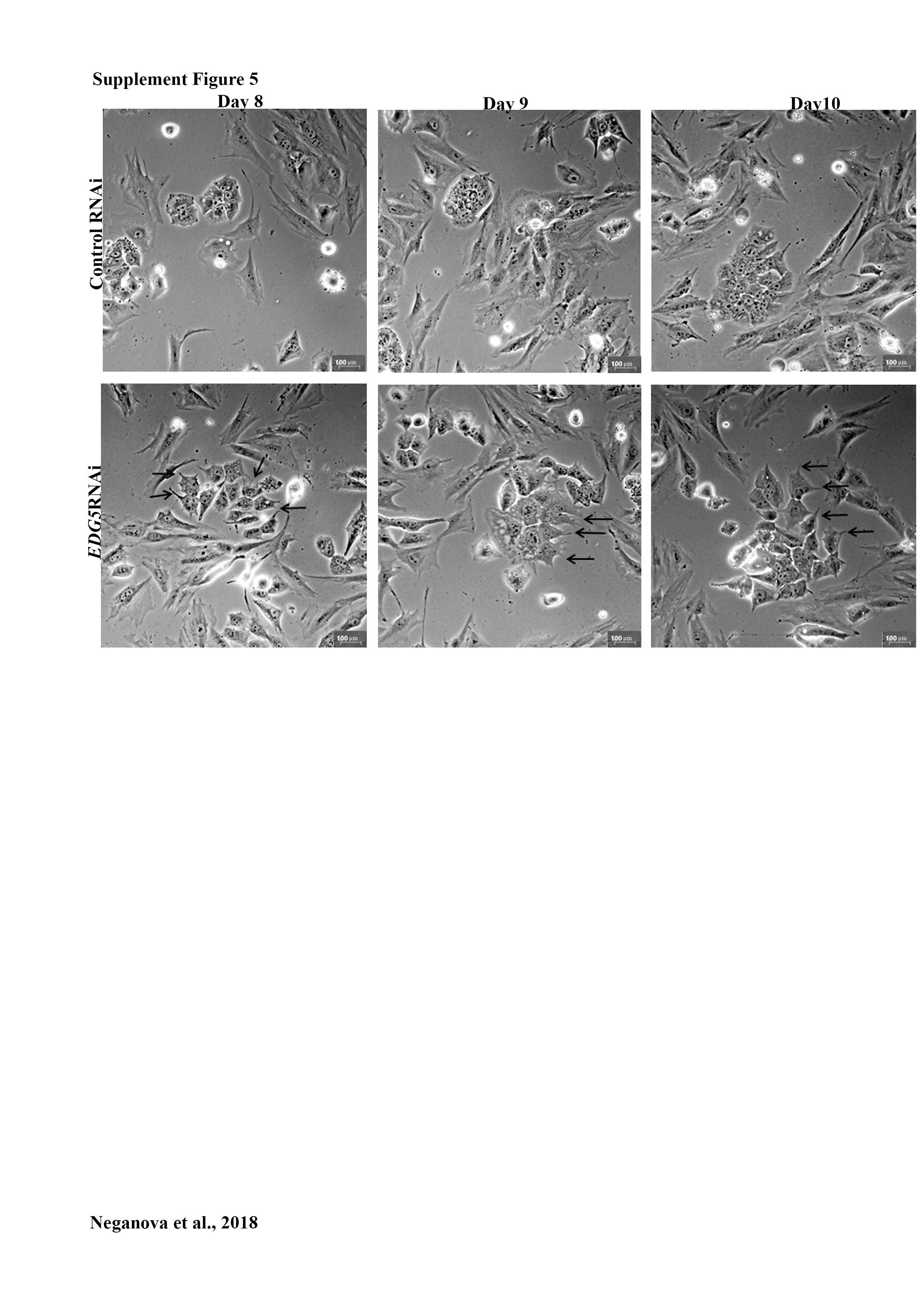

Supplement: Supplementary file 5 — Figure S5 EDG5 downregulation alters the typical morphology of the emerging hiPSCs colonies. (A) Representative bright‐field images of the Control and EDG5 siRNA treated groups during OSKM reprogramming from day 8 till day 10. Black arrows point to the numerous projections in EDG5 siRNA treated RNAi cells. Note sparse distribution and absence of the define edges in EDG5 siRNA treated RNAi, n = 3. Scale bar 100um. [file STEM-37-318-s005.jpg]
