# Supplementary material for: Endothelial Differentiation G Protein‐Coupled Receptor 5 Plays an Important Role in Induction and Maintenance of Pluripotency
Source: Stem Cells. 2019 Feb 7;37(3):318–31. doi: 10.1002/stem.2954 (PMC6446721; doi:10.1002/stem.2954)
Supplement: Supplementary file 6 — Supplement Table 1: XXX. [file STEM-37-318-s006.docx]

**Supplement Table 1**

| **Gene** | **Forward primer sequence (5’→3’)** | **Reverse primer sequence (5’→3’)** |
| --- | --- | --- |
| *EDG5* | TCATCCTCTGTTGCGCCATT | *ACAGGTACATTGCCGAGTGG* |
| *OCT4* | GAGAACCGAGTGAGAGGCAACC | CATAGTCGCTGCTTGATCGCTTG |
| *NANOG* | AATACCTCAGCCTCCAGCAGATG | TGCGTCACACCATTGCTATTCTTC |
| *KLF4* | TTACCAAGAGCTCATGCCACC | GCGAATTTCCATCCACAGCC |
| *SOX2* | TTGTTCGATCCCAACTTTCC | ACATGGATTCTCGGCAGACT |
| *GATA4* | TCCAAACCAGAAAACGGAAG | AAGGCTCTCACTGCCTGAAG |
| *LEFTY1* | AGAGCTGGCGATGACTGAAC | AAACTGAGCAAGGGCTCTCC |
| *EOMES* | CCAGGTTCTGGCTTCCGTG | TACATTTTGTTGCCCTGCATGTT |
| *NESTIN* | GAGAGGGAGGACAAAGTCCC | CCACTTCCTCAGACTGCTCC |
| *MIXL1* | GAGACTTGGCACGCCTGT | GGTACCCCGACATCCACTT |
| *NODAL* | GTACATGCTGAGCCTCTACCG | CAAAAGCAAACGTCCAGTTCTG |
| *SNAIL1* | TAGCGAGTGGTTCTTCTGCG | AGGGCTGCTGGAAGGTAAAC |
| *ZEB1* | CCACACGACCACAGATACGG | CCTGAGGAGAACTGGTTGCC |
| *TWIST* | GGAGTCCGCAGTCTTACGAG | TCTGGAGGACCTGGTAGAGG |
| *VIMENTIN* | GAGAACTTTGCCGTTGAAGC | GCTTCCTGTAGGTGGCAATC |
| *E-CADHERIN* | TGCCCAGAAAATGAAAAAGG | GTGTATGTGGCAATGCGTTC |
| *GAPDH* | TGCACCACCAACTGCTTAGC | GGCATGGACTGTGGTCATGAG |

SMARTpool: siGENOME siRNA ***EDG5*** ( Dharmacon, M-004253-02 )

D-003952-05 EDG5 9294 NM_004230 134244586 CCAACAAGGUCCAGGAACA

D-003952-09 EDG5 9294 NM_004230 134244586 GACAAGAGCUGCCGCAUGC

D-003952-10 EDG5 9294 NM_004230 134244586 GCCAAUACCUUGCUCUCUG

D-003952-11 EDG5 9294 NM_004230 134244586 UUGCCAAGGUCAAGCUGUA
